# The Human Cost of Anthropogenic Global Warming: Semi-Quantitative Prediction and the 1,000-Tonne Rule

**DOI:** 10.3389/fpsyg.2019.02323

**Published:** 2019-10-16

**Authors:** Richard Parncutt

**Affiliations:** Centre for Systematic Musicology, University of Graz, Graz, Austria

**Keywords:** anthropogenic climate change, carbon dioxide, mortality, ethics, order-of-magnitude

## Abstract

Greenhouse-gas emissions are indirectly causing future deaths by multiple mechanisms. For example, reduced food and water supplies will exacerbate hunger, disease, violence, and migration. How will anthropogenic global warming (AGW) affect global mortality due to poverty around and beyond 2100? Roughly, how much burned fossil carbon corresponds to one future death? What are the psychological, medical, political, and economic implications? Predicted death tolls are crucial for policy formulation, but uncertainty increases with temporal distance from the present and estimates may be biased. Order-of-magnitude estimates should refer to literature from diverse relevant disciplines. The carbon budget for 2°C AGW (roughly 10^12^ tonnes carbon) will indirectly cause roughly 10^9^ future premature deaths (10% of projected maximum global population), spread over one to two centuries. This zeroth-order prediction is relative and in addition to existing preventable death rates. It lies between likely best- and worst-case scenarios of roughly 3 × 10^8^ and 3 × 10^9^, corresponding to plus/minus one standard deviation on a logarithmic scale in a Gaussian probability distribution. It implies that one future premature death is caused every time roughly 1,000 (300–3,000) tonnes of carbon are burned. Therefore, any fossil-fuel project that burns millions of tons of carbon is probably indirectly killing thousands of future people. The prediction may be considered *valid*, accounting for multiple indirect links between AGW and death rates in a top-down approach, but *unreliable* due to the uncertainty of climate change feedback and interactions between physical, biological, social, and political climate impacts (e.g., ecological cascade effects and co-extinction). Given universal agreement on the value of human lives, a death toll of this unprecedented magnitude must be avoided at all costs. As a clear political message, the “1,000-tonne rule” can be used to defend human rights, especially in developing countries, and to clarify that climate change is primarily a human rights issue.

## Introduction

Anthropogenic global warming (AGW) is a human rights issue (Amnesty International, n.d.; Caney, 2010). It is violating the rights of future people—especially, in developing countries that will suffer the most. Lancet Countdown on health and climate change has warned that “A rapidly changing climate has dire implications for every aspect of human life, exposing vulnerable populations to extremes of weather, altering patterns of infectious disease, and compromising food security, safe drinking water, and clean air” (Watts et al., 2018). UN Environment (2019) found that “nearly one quarter of all deaths globally in 2012 could be attributed to modifiable environmental risks, with a greater portion occurring in populations in a vulnerable situation and in developing countries” (p. 22). From a legal perspective, “a right to a healthy environment in various formulations is recognized by the constitutions of 118 nations around the world” (Kravchenko, 2007, p. 539).

Progress toward global emissions reductions has been consistently slow (Ge et al., 2019). Contrary to the primary aim of the United Nations Climate Change Conferences held yearly since 1995, emissions increased by 2.2% per year on average between 2005 and 2015 (Le Quéré et al., 2018) and peaked again in 2018 (International Energy Agency, 2019). The current rate of carbon emissions is some 10 times greater than the last time global mean surface temperature (GMST) was relatively high, 56 million years ago (Gingerich, 2019). AGW has therefore become a global emergency (Ripple et al., 2017).

In responding to this challenge, it may help to express the urgency in new terms by shifting attention from economic to human costs, which are incomparably greater (Nolt, 2011a, 2015). The aim of this contribution is to defend the human rights of present and future people from the fatal indirect consequences of AGW caused by greenhouse gas (GHG) emissions and AGW by addressing the quantitative relationship between fossil carbon burned now and future deaths attributable to AGW.

The broader context involves interculturality and anti-racism research. The failure of rich countries and corporations to adequately mitigate AGW is racist in the sense that the protagonists are mainly white and the victims are mainly black (cf. Kaijser and Kronsell, 2014). AGW may also be considered sexist, given known gender differences in effects of AGW on health and life expectancy (World Health Organisation, 2011). AGW is ageist in that the emissions of today’s older people will disproportionately affect today’s young people (Page, 1999).

How much fossil carbon must be burned to cause a future human death? Despite the inherent uncertainties, it is interesting to attempt a zeroth-order estimate, based on semi-quantitative considerations of the current state of global climate, the current global rate of emissions, and the complex, non-linear relationships among the amount of carbon burned, corresponding changes in GMST, current mortality in connection with poverty, and future death tolls. The question is explicitly interdisciplinary: it involves humanities (e.g., philosophy, history), sciences (e.g., physics, mathematics, statistics, psychology), practically oriented disciplines (e.g., law, medicine, international development), and disciplines that mix these groups (economics, sociology). “The greatest potential for contributions from psychology comes not from direct application of psychological concepts but from integrating psychological knowledge and methods with knowledge from other fields of science and technology” (Stern, 2011, p. 314).

Of all the living and non-living things that humans encounter in their everyday lives, human lives are usually considered the most valuable (Harris, 2006)—regardless of the assumed value of non-human life (Kellert, 1997). Moreover, people are universally considered inherently more important than money (cf. Sayer, 2011); this general idea holds even if a human life can be assigned monetary value corresponding to the amount that others are willing to pay to save it. The value of a quality-adjusted life year (QALY) according to this criterion may effectively be of the order of $100,000 (Hirth et al., 2000). Can the continued use of fossil fuels be justified after comparing today’s health and longevity benefits with future health and longevity deficits due to AGW?

The following text begins with a summary of ways in which AGW will shorten human lives in the future. The idea of a human life as a mathematical unit of value is then introduced. After a consideration of the use of numbers and words in public discourse on AGW, and the psychological mechanisms that might distort estimates of future death tolls, an approximate top-down estimate is presented for the relationship between carbon burned now and deaths caused in the future. Ethical and political implications are addressed.

## How Anthropogenic Global Warming will Cause Premature Deaths

Historically, burning carbon has had a large positive effect on human life expectancy and quality of life (Steinberger and Roberts, 2010; Jorgenson, 2014). Without explicitly considering AGW, United Nations (2017b) estimated that from 1960 to 2100, global mean life expectancy will have increased from 46 to 83 years, among other things due to increasing availability of energy for agriculture, heating, cooking, transport, manufacture, and construction.

But carbon-based economies are also causing life-years to be lost in the future. The political challenge, therefore, is to maintain increases in life expectancy due to industrialization while minimizing losses in life expectancy due to AGW by replacing carbon-based power sources by sustainable ones.

The following brief summary of widely accepted climate impact predictions illustrates the magnitude of the problem:

1. Rising seas will threaten coastal homes and cities. Salination of agricultural soils will destroy farming land.
2. Dry areas will become drier with longer droughts, loss of ground water, and deglaciation. Agriculture will be seriously affected.
3. Serious storms (hurricanes, cyclones, and tornadoes) will become more frequent and dangerous (Knutson et al., 2015), destroying crops and buildings, and causing floods and epidemics (cf. the cholera outbreak that followed Cyclone Idai in Mozambique in 2019; Nguyen et al., 2019).
4. Heat waves will become more frequent and intense. When wet-bulb temperatures approach human skin temperature, body temperature can no longer be regulated by perspiration—with fatal consequences.
5. The current rate of species extinction (biodiversity loss)—already 100–1,000 times faster than without humans—will continue to increase (sixth mass extinction event).

Each of these points will affect supplies of food and fresh water, increasing current death rates due to hunger and disease. In addition, AGW will affect the nutritional content of staple crops such as rice and wheat; when carbon dioxide (CO_2_) levels double relative to pre-industrial levels, an additional 175 million people may be zinc deficient; 122 million, protein deficient (Smith and Myers, 2018). These points may interact with each other, causing ecological cascade effects and co-extinctions. AGW will also increase the incidence and magnitude of international conflicts including water wars (Petersen-Perlman et al., 2017).

There is an additional risk of “runaway” AGW, in which GMST continues to rise after anthropogenic emissions stop—driven by natural positive feedback processes that are not canceled by negative ones:

1. When ice melts, less radiated heat from the sun is reflected back into space, so more is absorbed, causing more ice to melt (Albedo).
2. As the carbon content of oceans and soils increases, their ability to absorb CO_2_ falls (Gattuso et al., 2015).
3. When permafrost (tundra) peat thaws, it releases CO_2_, methane (CH_4_), and nitrous oxide (N_2_O), causing more warming and melting (Voigt et al., 2017). Permafrost peat contains about 1,700 Pg carbon—about twice as much as the entire atmosphere—of which 30% (68–508 Pg) could be released by 2100 (MacDougall et al., 2012). Atmospheric CH_4_ concentration has unexpectedly accelerated in recent years (Nisbet et al., 2018).
4. Forests will dry out at the same time as weather conditions that cause fires (dry soil, high temperature, low humidity, and high winds) become more frequent. Fires produce CO_2_, causing more warming and drying (Gabbert, 2018; Reidmiller, 2018). Forest dieback can be caused by a combination of drought and bark-beetle infestation, caused in turn by AGW (Sangüesa-Barreda et al., 2015). Beetle-caused dieback can switch a forest from a carbon sink to a carbon source (Hansen et al., 2013a). Between 1984 and 2016, the European forest area affected by mortality doubled—largely due to AGW and land-use changes (Senf et al., 2018).
5. Extreme temperatures caused by climate change will increase human energy consumption for heating and cooling (International Energy Agency, 2019).

When feedbacks are taken into account, the global carbon budget for limiting AGW to 2 or 1.5°C is reduced by “several years of anthropogenic carbon dioxide emissions at present rates” (Lowe and Bernie, 2018, abstract).

## The Human Life as a Unit of Value

Consider the following two theses: (1) human lives are equal in value and (2) human lives are the most valuable thing that humans know. Scientific research is consistent with (1), having failed to find evidence for inherent biological or cultural differences in value or ability (e.g., intelligence) between human groups (Fairchild, 1991). Consistent with both points, Kant (1785/2011) proposed a “kingdom of ends,” in which people are always considered as ends and never as means, implying that their value is incomparable with other forms of value.

The two theses have a strong legal foundation. The Universal Declaration of Human Rights, adopted by the United Nations in 1948, repeatedly refers to human equality. The first sentence of the declaration specifies “the inherent dignity and of the equal and inalienable rights of all members of the human family.” The preamble also mentions the “equal rights of men and women.” Article 1 proclaims that “All human beings are born free and equal in dignity and rights.” Article 7 adds that “All are equal before the law and are entitled without any discrimination to equal protection of the law.” According to Article 10, “Everyone is entitled in full equality to a fair and public hearing by an independent and impartial tribunal.” The principle of equality also applies to marriage (Article 16), access to public service and voting rights (21), pay for work (23), and access to education (26).

This background justifies using the human life as a unit for measuring the size of a disaster or catastrophe—comparable with kilograms, meters, and seconds for physical measurements. The number of deaths associated with a given event is an objective (although approximate) measure of the suffering associated with that event, and hence with its magnitude or seriousness.

Subjective estimates are different. First, the perceived size of a number is not proportional to its actual size: “one billion” does not seem a thousand times bigger than “one million”. Second, psychic numbing (Lifton, 1982) means a large disaster may not seem bigger or more important than a small one. Neuroscientific evidence (Dehaene, 2003) points to an approximately logarithmic relationship between the number of deaths and the perceived magnitude of a disaster.

A scientifically founded humanitarian approach should aspire to overcome such subjective limitations. “This perspective presumes a linear relationship between the number of lives one can save in a given situation and the value associated with saving them. Thus an effort saving 200 lives would have twice the value of another that saves 100 lives” (Fetherstonhaugh et al., 1997, p. 285).

## Estimating Future Death Tolls

Future death tolls in connection with AGW will depend on climate in various ways. Changes in extreme temperatures (lows in winter and highs in summer) at a given location are one of many possible climate-related causes of death; studies that focus on this aspect (e.g., Kalkstein and Greene, 1997; Nicholls, 2009; Huber et al., 2017) may be ignoring the main drivers. Death rates will be highest in developing countries or among people living in poverty, so studies with that focus are more relevant.

AGW interacts in complex ways with several of the Sustainable Development Goals (Nilsson et al., 2016). Many different climate impacts could directly or indirectly lead to premature death or exacerbate existing rates of premature death from hunger or avoidable disease. Vector- and rodent-borne diseases including arboviral (dengue, chikungunya, West Nile, and malaria) may change their geographic distribution with climate change (temperature, extreme weather events, and seasonality) and environmental factors (land-use, ecosystems, deforestation, hydrology, and biodiversity); rodent population density and distribution are also affected by weather conditions (Apfel, 2007, p. 4). “(H)uman illnesses due to antimicrobial-resistant infections may become a major cause of death from infectious diseases worldwide by 2050” (UN Environment, 2019, p. 12). At the same time, food demands may increase by 50% (Searchinger et al., 2018).

The positive effect of international development projects on global rates of hunger may recently have been overtaken by the negative effect of AGW. In 2015, the proportion of undernourished people on each continent varied between 7 and 19%; the world average was 11% in 2016 (Our World in Data, n.d.). This proportion decreased steadily in recent decades on all continents. But in low-income countries, the proportion of undernourished people was 27.2% in 2015, 28.2% in 2016, and 28.3% in 2017. Roser and Ritchie (2018) explained:

This increase in hunger levels is largely a result of increases in Sub-Saharan Africa (where rates have risen by several percentage points in recent years) and small increases in South America (from 4.7% in 2014 to 5% in 2017). The UN FAO have linked this increase in undernourishment in particular to the rising extent of conflict-affected countries (which is often a leading cause of famine), and compounded by climate-related factors such as the El Niño phenomenon (which can inflict both drought and flood conditions).

Future death tolls due to AGW will also involve conflicts such as water wars (Gleick, 2014). Hence, both reasons for the recent increase in hunger offered by Roser and Ritchie involve climate.

DARA International (2012) linked 400,000 annual deaths worldwide to AGW. World Health Organisation (2017) found that “Between 2030 and 2050, climate change is expected to cause approximately 250,000 additional deaths per year, from malnutrition, malaria, diarrhea and heat stress.” These estimates seem conservative when considered relative to existing death rates in connection with poverty. Three million children are still dying of hunger every year (Black et al., 2013). AGW is already affecting food and fresh water supplies, increasing the death rate from hunger (McMichael et al., 2006, 2008). Many deaths are caused by a combination of poverty and AGW.

World Health Organization (2009) explained that “Over time, major risks to health shift from traditional risks (e.g. inadequate nutrition or unsafe water and sanitation) to modern risks (e.g. overweight and obesity). Modern risks may take different trajectories in different countries, depending on the risk and the context” (p. 3). From a global perspective, traditional risks have been declining, whereas modern risks are increasing. But AGW is causing traditional risks to rise again relative to modern risks, and predictions of the Intergovernmental Panel on Climate Change (IPCC) in different scenarios suggest that this trend will continue until 2100.

The death rate from hunger will increase not only due to AGW itself but also due to efforts to mitigate and adapt to AGW:

Food insecurity can be directly exacerbated by climate change due to crop-production-related impacts of warmer and drier conditions that are expected in important agricultural regions. However, efforts to mitigate climate change through comprehensive, economy-wide GHG emissions reductions may also negatively affect food security, due to indirect impacts on prices and supplies of key agricultural commodities (Hasegawa et al., 2018, p. 699).

Climate Change Impacts and Risk Analysis (2015) found that a global agreement to reduce AGW could prevent 70,000 premature American deaths annually by 2100. But death rates in developing countries will be much higher, especially when infections, parasites, AIDS, diarrhea, tuberculosis, malaria, and childhood diseases are considered. Predictions for richer countries do not tend to focus on the combined effects of poverty, hunger, and water supplies, but instead on factors such as air quality, extreme temperatures, water quality, extreme weather events, air pollution from wildfires, and vector-borne disease.

In a media discussion over a decade ago, journalist George Monbiot commented that “If we don’t deal with climate change we condemn hundreds of millions of people to death” (Democracy Now, 2007). Given current extrapolations and speculations about future poverty, hunger, disease, migration, and war, this top-down estimate seems reasonable. Nolt (2011a) found that the emissions of a typical US-American over the course of a lifetime cause the suffering or death of one or two future people in poor countries during the next millennium. Of course, not only Americans are to blame, although their emissions are roughly twice those of Europeans; in round figures, 10^9^ rich people are in the process of prematurely ending the lives of 10^9^ future poor people. Nolt (2015) summarized other attempts to estimate future death tolls in connection with AGW, concluding that “the methodologies of these studies are imperfect … clearly there is need for critique and further refinement of such estimates” (p. 353).

Summarizing this section, existing estimates of future AGW death tolls suffer from various limitations. Many focus on mortality due to extreme temperatures, but mortality from poverty combined with AGW could be much higher. Many focus on rich countries, detracting attention from the more serious plight of developing countries. Most are limited to the next few decades, but the problem will probably become more acute every decade until at least 2100, given that the half-life or atmospheric lifetime of anthropogenic CO_2_ in the atmosphere is of the order of a century (Archer et al., 2009).

### Reliable Versus Valid Prediction of Future Death Tolls

Existing predictions of future AGW death tolls are *bottom-up* in two senses: *analytic*, considering deaths from individual causes in separate regions or countries, and *empirical*, based on past experience. Conversely, a *top-down* estimate is *holistic*, considering the entire global population, and *extrapolatory*, considering unprecedented future developments. A top-down estimate is *inductive*, beginning with an initial guess that is refined after comparison with sources of evidence; it mixes *hermeneutics* (humanities) with *active inference* (psychology; Friston and Frith, 2015).

By analogy, psychological measures and tests can be *reliable* or *valid*. A reliable test gives a similar result on different occasions. A valid test tests what it is intended to test and not something else (internal validity) and is generalizable beyond a given sample (external validity) (Drost, 2011). A reliable test is not necessarily valid, and vice-versa.

A bottom-up analysis of future deaths attributable to AGW has the advantage of reliability based on locally relevant details and documented past experience. In Bangladesh, for example, DARA International (2012) predicted 2,600 annual deaths from environmental disasters and 20,000 from various diseases attributable to AGW per year by 2030. But a top-down analysis that attempts to see the big picture while extrapolating current trends may be more valid, better estimating what it intends to estimate (the death toll in a future unprecedented situation), attempting to generalize non-linearly beyond a limited sample, and including the highly uncertain possibility of unprecedented, catastrophic developments. A top-down analysis may produce a more realistic grand total but lack a detailed breakdown.

For these reasons, a more rigorous multivariate analysis that considers relevant territorial, geographic, population, health, epidemiological, economic, and geopolitical aspects of the problem will not be attempted here. Instead, I will present a big-picture, top-down estimate. Nor will I attempt a separate consideration of IPCC emissions scenarios (rapid economic growth; global environmental sustainability; regionally oriented economic development; local environmental sustainability; Nakicenovic et al., 2000) or the Representative Concentration Pathways (different levels of radiative forcing in the year 2100; Van Vuuren et al., 2011). My focus will instead be on a single scenario in which GMST rises to 2°C above pre-industrial temperatures. Limiting calculations to order-of-magnitude estimates (OMEs) means that mortality predictions based on different 2°C scenarios—different global-emission or CO_2_-concentration trajectories that lead to the same maximum GMST—are unlikely to differ. Moreover, by contrast to IPCC scenarios, my approach includes the possibility of highly uncertain outcomes such as climate-based wars and catastrophic ecological events.

As an example of highly uncertain outcomes, consider the effect of AGW on displaced persons. The Environmental Justice Foundation warned that AGW could create 150 million climate refugees in the next 40 years (Guardian, 2009), or 3 to 4 million per year. Many climate refugees will die before finding a new home. Current EU policy on refugees from Africa, driven by the rise of the political far right, suggests that the average European citizen is more concerned about stopping illegal immigration than preventing deaths at sea; “non-Europeans from poor countries seeking entry into Italy are categorized as outsiders and therefore non-human” (Carter and Merrill, 2007). Future far-right governments, having consolidated power by undermining democratic infrastructures, will explore new ways of preventing immigration (Jones, 2016)—possibly with fatal consequences for enormous numbers of people.

## Temporal Issues

An estimate of the total death toll due to AGW should consider the period during which deaths will occur relative to human life expectancy. Current emissions will probably cause future deaths for between a century and a millennium. CO_2_ emissions (mainly from fossil fuels) stay in the atmosphere for 2–20 centuries (Walker, 1985; Archer et al., 2009); CH_4_ (mainly from ruminant livestock), for about decade (Prather et al., 2012). The third most important anthropogenic GHG, N_2_O, has an atmospheric half-life of roughly a century and is emitted by agricultural soils, tropical soils, and melting permafrost (Wilkerson et al., 2019). If all anthropogenic emissions suddenly stopped, half the anthropogenic CO_2_ would disappear in the following decades, the duration depending on the state of the world’s oceans and forests; a smaller fraction would remain for thousands of years. It is possible that the effects of AGW will continue for tens of thousands or even a hundred thousand years (Solomon et al., 2009; Zeebe, 2013).

Another temporal issue is the relationship between *lives lost* and *years of life lost*. Years of life lost are used in medicine to evaluate the impact of a disease (e.g., Fontaine et al., 2003) and suggest that that the death of a child more serious than the death of an adult.

Without AGW, the life expectancy of a child born today in a developing country depends on country, varying from 35 to 70 years (Central Intelligence Agency, n.d.). Of those children and adults that die in connection with AGW in coming decades or centuries, the average number of life-years they will lose can only be guessed. The many negative consequences of AGW and the special vulnerability of the very young (Black et al., 2013) and the very old (Carter et al., 2016) suggest that the age distribution of future victims of climate change will be bimodal. In a zeroth-order estimate, an average future AGW-victim in a developing country might lose half of a lifetime or 30–40 life-years. An additional factor is the population pyramid (Cohen, 2003).

When disability is taken into account (*disability-adjusted life years* or DALYs; Murray, 1994), the rate at which life years are effectively lost depends on the severity of the illness or injury. But DALYs have an important practical disadvantage:

The public does not understand them. If to the question, “how much harm will climate change do in the next decade (or century)” one answers with a certain large number of DALYs lost, this will mean nothing to most people. If, on the other hand, one answers with a casualty or fatality estimate, nearly everyone will understand. (Nolt, 2015, p. 351).

Nolt (2015) listed three advantages of limiting the discussion to simple numbers of deaths: such estimates are empirical (based on hard facts), universally understood, and “more likely … to elicit constructive public concern” (p. 353). After comparing deontological (duty- or rule-based) and consequentialist approaches to this problem, Nolt concluded that “casualty estimates are … significant no matter which ethical framework we use” (p. 354).

## Use of Clear, Direct Language

The consequences of climate change are more serious than the neutrally/positively connotated words “climate” and “change” suggest. In that sense, “climate change” may be considered a *euphemism*. AGW sounds more serious, although the words “anthropogenic,” “global,” and “warming” each sound neutral or positive. The apparently innocent word “adaptation” is problematic if its implementation leads to global social injustice (Kagawa and Selby, 2010).

Terminologies such as “lost life-years” and “climate and health” are euphemisms if they disguise or soften discourses that are really about killing or death (cf. Abbott, 2010). In everyday language, the word “kill” means to cause death by any means, direct or indirect, regardless of any associated intention. The word may be avoided or modified in scientific discourse to soften its illocutionary force (Holmes, 1984). But if the scientific questions being asked involve future people’s lives that are being shortened by AGW, or the effect of today’s consumerist, mobile lifestyles on future deaths, it is important to state the facts clearly. To protect the basic rights of children alive now or soon to be born, living in poverty in developing countries such as Congo, Liberia, Zimbabwe, Afghanistan, or Haiti, we must directly and openly consider the mechanisms that might cause their deaths.

Conversely, exaggeration should be avoided. Sociologist and human rights activist Jean Ziegler claimed that every child who dies of hunger is “murdered” (United Nations, 2002). He was right to attract attention to a very serious problem. But in everyday and legal usage, “murder” involves premeditated malicious intention, whereas “manslaughter” does not. The politicians and corporate CEOs most responsible for child mortality through hunger do not intend to kill anyone.

For similar reasons, it is problematic to compare AGW with genocide, and Nazi Holocaust comparisons are rightly taboo (Maier, 1988). The Holocaust was unique due to the deliberate and premeditated nature of the killing (Sweeney, 2012). AGW is not about killing specific people. The identity of future victims can be predicted only approximately: they are more likely to live in developing or tropical countries or high-risk regions. The timeframe is similarly approximate (the coming century or two).

A quantitative human-rights perspective based on expected death rates enables the following comparisons. While the Nazi Holocaust was a greater *crime* than AGW due to its deliberate and premeditated nature, AGW will be a bigger *disaster* or *tragedy* if it causes ten or a hundred times more deaths. Both the number of lives lost per year and the duration of the crisis will be greater for AGW than for the Holocaust. Both the Holocaust and AGW are/were enabled by public indifference combined with active deception by powerful individuals in government or corporations. Historical, psychological, and sociological comparisons of this kind can help overcome both the indifference (Booth, 2012) and the deception (Sandman, 2009).

## Estimating Future Death Tolls

For the mathematical relationship between CO_2_ concentration and GMST, there is no single, widely accepted formula (Knutti et al., 2017). In an *exponential* approximation, GMST rises by a certain amount (equilibrium climate sensitivity) every time CO_2_ concentration doubles and stabilizes: roughly 3°C, or between 1.5 and 4.5°C (Myhre et al., 2017). In a *linear* approximation, the change in GMST (in °C or K) is proportional to the mass of carbon burned (Allen et al., 2009; Tokarska et al., 2016). The linear approximation takes into account carbon cycle feedbacks: as GMST increases, the ability of oceans and soils to absorb and store carbon decreases (Friedlingstein et al., 2006; Gregory et al., 2009). The resultant gradient of temperature rise as a function of carbon burned (transient climate response to cumulative CO_2_ emissions or carbon-climate response) is roughly constant at between 1 and 2°C per trillion (10^12^) tons of burned carbon. This is approximately true up to several trillion tons, or the end of reasonably accessible carbon reserves (Matthews et al., 2009). “(U)uncertainty in land-use CO_2_ emissions and aerosol forcing … means that higher observationally constrained values cannot be excluded” (p. 829), justifying the common estimate of 2°C per trillion tonnes^1^. Thus, burning 5 × 10^12^ tons (an estimate of all available fossil fuel) will cause roughly 10°C of warming (Tokarska et al., 2016).

Linear and exponential approaches make divergent predictions for several degrees of warming, but both are roughly consistent with the following prediction. When humanity burns a trillion tonnes of carbon (of which about half has been burned so far), GMST will rise by about 2°C altogether relative to pre-industrial times (the carbon budget; Harvey et al., 2013).

Given that the time period during which anthropogenic CO_2_ remains in the atmosphere is inherently uncertain—spanning decades, centuries, or even millennia—it is difficult to predict how many people will die prematurely as a result of 2°C of AGW. Consider the following radically simplified scenarios:

1. If the deaths were confined to the 21st and 22nd centuries, the AGW death rate during that period might vary between zero and the current global death rate in connection with poverty, which is roughly 10^7^ per year. Storms alone—more intense and frequent due to AGW—could cause millions of deaths per year. Given the multiple serious consequences of AGW and their separate and collective effects on food and water supplies, forced migration, and armed conflict, it is reasonable to assume the AGW will double the death rate in connection with poverty for a period of a century, causing 10^9^ deaths altogether. According to the World Bank, “By 2050, population growth and rapid urbanization could put 1.3 billion people and $158 trillion in assets at risk from river and coastal floods alone” (Phillips et al., 2018, p. 5).
2. If AGW increased the death rate by 50% for two centuries, the total number of deaths due to AGW would also be 10^9^. If the death rate was smaller (10^6^ per year) but continued for 10 centuries, the final death toll would again be 10^9^.
3. Combining the previous approaches, future death rates attributable to current and past emissions may vary between 10^6^ and 10^7^ per year for a period of several centuries. Mathematical integration of this kind is used in *risk assessment* (Henley and Kumamoto, 1981) and *expected value* calculations (Fankhauser, 1994). The expected value of the future death toll (a measure of risk) is calculated by adding products of estimated death rate and estimated probability for different scenarios.

**Figure 1:**
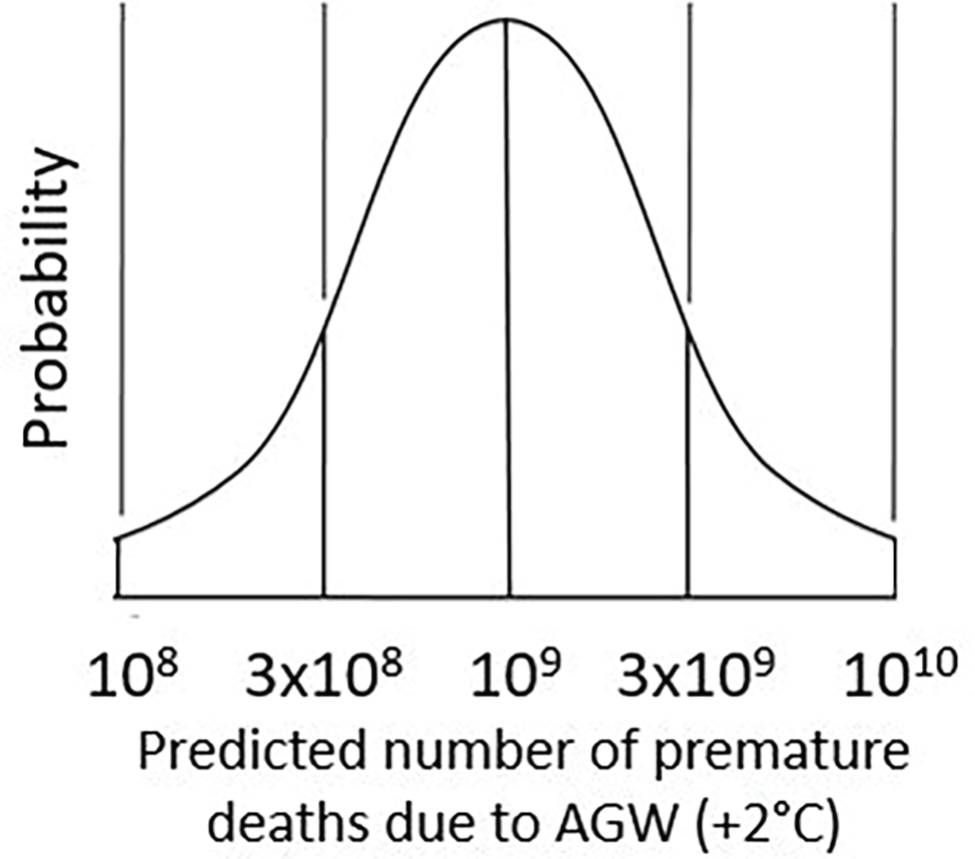
Assumed probability distribution of AGW-driven mortality. Predicted distribution of the total global number of premature deaths attributable to 2°C of AGW. 10^8^ is an extreme best-case estimate, 3 × 10^8^ is likely best case, 10^9^ is median, 3 × 10^9^ is likely worst case, and 10^10^ is extreme worst case.

Assuming a broad distribution of possible future death rates, a given death-rate estimate is plausible if considerably higher and lower estimates are also plausible. We should therefore consider best- and worst-case estimates before arriving at a most-likely estimate or expected value. In the following, a top-down approach will be presented in which the expected value of the total global death toll caused by 2°C AGW lies logarithmically midway between a likely best-case scenario of 3 × 10^8^ and a likely worst-case scenario of 3 × 10^9^.

These are no more than *order-of-magnitude estimates*. OMEs are commonly used in physics for approximate calculations involving very large or very small numbers (e.g., Serway and Jewett, 2018). The uncertainty of an OME is itself uncertain. Consider the number 1.36, correct to two decimal places or three significant figures. Depending on definition, this number lies either between 1.35 and 1.37 or between 1.355 and 1.365. Similarly, the order of magnitude “10^4^” lies either between 10^3^ and 10^5^ or between 10^3.5^ ≈ 3 × 10^3^ and 10^4.5^ ≈ 3 × 10^4^. The following argument assumes the smaller range (a factor of 3 in either direction).

## Best-Case Estimate

A likely best-case estimate of the future death toll due to 2°C AGW may be made by considering IPCC predictions for 2°C by comparison to 1.5° (Pirani et al., 2018). The long tradition of interdisciplinary peer-reviewed scientific research behind these predictions makes them *reliable*. Death rates predicted on the basis of IPCC data may be considered “best-case” if they ignore uncertainties involving interactions between different climate impacts, climate feedback processes, or “tipping points.”

Each of the following climate impacts listed by Masson-Delmotte and colleagues can be expected to contribute significantly to the AGW death toll at +2°C by comparison to 1.5°C:

1. direct weather impacts: higher extreme temperatures, longer heatwaves, more flooding and drought, more and more intense tropical cyclones;
2. oceans: higher sea level, temperature, acidity; dying coral reefs;
3. aquatic life: long-lasting or irreversible impacts on biodiversity and ecosystems including species loss and extinction, habitat, reproduction, disease, invasive species, and coral bleaching;
4. land: geographic range of insects, plants and vertebrates, degradation and loss of high-latitude tundra and boreal forests, thawing of permafrost;
5. for humans: effects on health, livelihoods, food security, water supply, security, economic growth; higher heat-related and ozone-related mortality; greater spread of vector-borne diseases (malaria, dengue fever); lower yields of maize, rice, wheat, soy; lower nutritional quality of rice and wheat; greater negative effects on livestock including changes in feed quality, spread of diseases, and water resource availability.

In a zeroth-order OME, each such point might be associated with a death toll of 10^6^ per year or 10^8^ per century that is attributable to AGW. A relatively optimistic, but also very approximate, best-case estimate of 3 × 10^8^ deaths is consistent with this approach if we also assume the success of diverse adaptive strategies in different geographic locations, depending on economic possibilities. Relevant adaptations might include smart housing, urban heat refuges, tree planting, water storage, desalination, medical/ecological suppression of vector-born disease, solar-powered air conditioning, flood defenses, agricultural innovations, and the creation of new habitats for endangered species. Given the lower adaptive capacity of poorer countries, adaption may also be promoted by alleviating poverty and inequalities and improving education; but the success of adaptive strategies is also limited by cultural factors (Adger et al., 2013).

Another approach is to consider existing death tolls from related causes. As a cause of death, AGW will hardly be inseparable from poverty, which is also linked to air pollution (Egondi et al., 2018). Outdoor air pollution (mostly by PM2.5) is now causing 3 × 10^6^ premature deaths per year, mainly in Asia; in a business-as-usual scenario, this figure could double by 2050 (Lelieveld et al., 2015). If the effect remained constant for a century, air pollution would kill 3 × 10^8^ people altogether. This estimate may be conservative: World Health Organisation (2018) estimated 4.2 × 10^6^ deaths per year due to ambient (outdoor) air pollution plus 3.8 × 10^6^ due to household cookstoves and fuels. UN Environment (2019) estimates that air pollution is causing 6 to 7 × 10^6^ deaths annually (1 in 9 deaths worldwide). About 91% of the world’s population lives in places, where air pollution exceeds WHO guideline limits.

A best-case scenario should also be optimistic about expected progress in coming years toward reducing the death toll from AGW. It should in particular assume improvements in:

1. technology, supported by sufficient finance, to sustainably improve food production and distribution in developing countries,
2. medicines and infrastructures to treat vector-borne diseases,
3. international diplomacy to promote the peaceful conflict resolution,
4. support services for climate refugees in transit and on arrival in new homelands, and
5. popular acceptance of refugees reflected in more liberal, internationally oriented government policies.

Taken together, these arguments are consistent with a best-case scenario in which AGW increases the current death rate in connection with poverty (roughly 10^7^ per year) by 30% to 1.3 × 10^7^ per year, the difference between the two rates (with and without AGW) remaining roughly constant for a century. The total death toll due to AGW of 2°C would therefore be 100 × (3 × 10^6^) = 3 × 10^8^.

## Worst-Case Estimate

A likely worst-case scenario should consider all reasonably possible catastrophic outcomes that might be caused directly or indirectly by AGW, even if limited to 2°C.

1. Today, about 30% of global population experiences deadly heat for over 20 days per year. By 2100, this will rise to 48% if GHG emissions are drastically reduced and 74% if they continue to grow (Mora et al., 2017).
2. The combination of AGW and high population growth in developing African countries such as Equatorial Guinea, Omar, Niger, Uganda, Angola, and Congo will lead to unprecedented death rates due to poverty (hunger, disease, and violence) and massive population displacement. Africa’s population (currently 1.3 × 10^9^) will rise to roughly 2.5 × 10^9^ by 2050 and 4 × 10^9^ by 2100^2^. Between 2017 and 2050, 26 African countries may double their populations (United Nations, 2017b). Even without AGW, it will not be possible to produce and deliver sufficient food and fresh water (Godfray et al., 2010). AGW will exacerbate the crisis—even without considering population growth (McMichael et al., 2006, 2008). By 2100, the total death toll due to 2°C AGW may approach 10^9^ in Africa alone.
3. There will be severe climate impacts in the Middle East and Northern Africa, with mean temperature increases well above GMST and displacement of large human populations (Economist, 2018).
4. Thomas et al. (2004) estimated that 15% of all species will be extinct by 2050 if AGW is limited to 1.5°C; 37% if limited to 2°C. Ecological dependencies may multiply the direct effects of environmental change on the collapse of planetary diversity by 10 (Strona and Bradshaw, 2018). Loss of biodiversity will make it impossible to feed a larger African population (Frison et al., 2011).
5. Insect populations will be affected by a combination of AGW and insecticides (Boggs, 2016). Forty percent of the world’s insect species may go extinct in coming decades (Resnick, 2019; Sánchez-Bayo and Wyckhuys, 2019). In the past 50 years, bee pollinations have declined as demand for agricultural pollination has approximately tripled, triggering a pollination crisis that affects crop yields (Goulson et al., 2015). Extinction of bee species could lead to the extinction of plant species that depend on bees for pollination, leading to other animal, plant, and insect extinctions, which in turn affects insect-eating bird populations (Goulson, 2014).
6. Biodiverse coral reefs will be degraded due to pollution, overfishing, and rising temperature and acidity of ocean waters (Hughes et al., 2017). Oceanic oxygen concentration is also falling (Poppick, 2019). Most reefs will be seriously threatened or irreversibly damaged by 2050 (Burke et al., 2011) and the rest may die by 2100.
7. Sekerci and Petrovskii (2015) showed that “the oxygen production by marine phytoplankton can stop suddenly if the water temperature exceeds a certain critical value. Since the ocean plankton produces altogether more than one half of the total atmospheric oxygen, it would mean oxygen depletion not only in the water but also in the air. Should it happen, it would obviously kill most of life on Earth” (p. 2349).
8. Soil will be degraded by chemical-heavy farming techniques and deforestation-induced erosion, reducing crop yields (Arsenault, 2014). “There is rapidly escalating competition between the demand for land functions that provide food, water, and energy, and those services that support and regulate all life cycles on Earth” (United Nations, 2017c, p. 8).
9. Groundwater (Dalin et al., 2017) is the largest available store of global freshwater and 2 × 10^9^ people rely on it. About 6% of global groundwater is readily available and can be replenished with a human lifespan (Gleeson et al., 2016). Where groundwater is depleted, recovery may take centuries or millennia (Cuthbert et al., 2019, p. 140).
10. About 10% of global land is covered by glaciers, and 10^9^ people depend on their meltwater (Qui, 2019). AGW will cause non-polar glacier volume to fall by 29–41% in 2100; “glaciers in Central Europe, low-latitude South America, Caucasus, North Asia, Western Canada, and US are projected to lose more than 80% of their volume by 2100” with “major implications for regional hydrology and water availability in the near future” (Radić et al., 2014). Clarke et al. (2015) predicted that by 2100 Western Canadian glaciers will shrink by 70% relative to 2005, affecting aquatic ecosystems, agriculture, forestry, and water quality. Iceland’s glaciers will shrink by 40% in 2100 and 100% in 2200 (Poore et al., 2000). Accelerated deglaciation in Greenland from 2003 to 2013 suggests a tipping point driven by changes in air temperature and solar radiation (Bevis et al., 2019).
11. Pathogens such as anthrax may emerge from melting permafrost (Revich and Podolnaya, 2011; Legendre et al., 2015) and cause regional or global pandemics (Wu et al., 2016). Humans have little immune resistant to zoonoses—diseases transmitted between human and non-human animals, such as ebola and salmonellosis. In the 14th century, bubonic plague (spread by fleas or body fluids from plague-infected animals) killed 25–40% of European children and adults (Galvani and Slatkin, 2003).
12. 2°C AGW will trigger conflicts over natural resources (Barnett and Adger, 2007). Political destabilization could lead to use of nuclear weapons, causing radioactive fallout and ozone depletion (Mills et al., 2008).

In a zeroth-order estimate, one or more of these points could alone cause 10^7^ deaths per year for a century—a total of 10^9^ deaths each. If that is true, a worst-case estimate of 3 × 10^9^ for the worst-case AGW death toll may be realistic. That would correspond to roughly 30% of future world population, which will reach 9.8 × 10^9^ in 2050 and 11.2 × 10^9^ in 2100 (or between 8 × 10^9^ and 15 × 10^9^; United Nations (2017a). Given the high degree of uncertainty, a more precise estimate is hardly realistic.

Incorporating the above best- and worst-cases into a simple mathematical model, the probability distribution of different possible global mortality outcomes may be normal (Gaussian) relative to a logarithmic axis, as shown in Figure 1. This distribution is no more than an educated guess or top-down estimate based on the following assumptions. Approximately 2/3 of possible outcomes lie within one standard deviation of the mean and 95% within two standard deviations. The number of premature deaths attributable to AGW (when limited to +2°C) may lie between 3 × 10^8^ and 3 × 10^9^ (*likely* best- and worst-case outcomes) with a probability of 2/3, and between 10^8^ and 10^10^ (*extreme* best- and worst-case outcomes) with a probability of 95%. Regarding the lower limit of 10^8^, even if GMST suddenly stabilized at +1°C, we might reasonably predict 10^6^ deaths per year for a century due to AGW, making the 10^8^ estimate obviously too low. The upper limit of 10^10^ is obviously too high: in the absence of unexpected climate feedbacks or tipping points, the probability that 2°C AGW will kill most or all humans is negligible.

## Visualizing the Unfolding Global Tragedy

**Figure 2:**
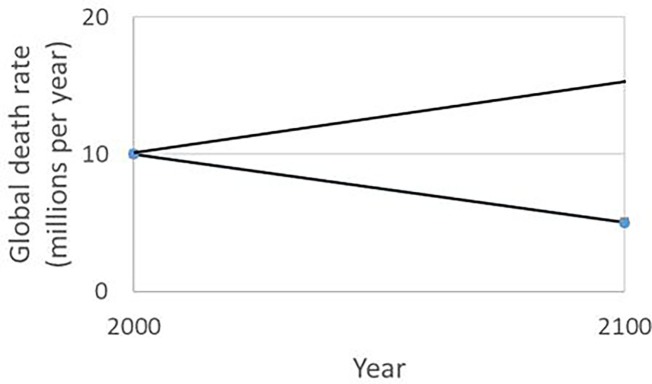
Predicted development of global mortality due to poverty and AGW. Very approximate projection of global annual death rates during the 21st century. The lower line represents deaths due to poverty without AGW. As the negative effect of AGW overtakes the positive effect of development, the death rate will increase, as shown by the upper line. In a more accurate model, the upper line might be concave upward on the left (exponential increase) and concave downward on the right (approaching a peak).

Figure 2 is a conceptual sketch of the death rate in connection with global poverty and AGW as it might develop during the 21st century. Again, the graph is no more than an educated guess or top-down estimate based on a small number of holistic assumptions. The lower line is a rough estimate of the projected death rate in connection with poverty (hunger, curable disease, preventable disease, violence, and air pollution) in developing countries. Depending on assumptions and operationalizations, this rate is currently roughly 10^7^ per year. It has been gradually falling for decades, due to economic growth combined with the efforts of developmental aid organizations, partly in the framework of the Millennium Development Goals and Sustainable Development Goals of the United Nations (Sachs, 2008).

Without AGW, the lower line of Figure 2 would continue to descend. But the emerging combination of poverty and AGW means it will probably rise in coming decades (McMichael et al., 2006, 2008). Given the multiple serious effects of AGW on global food and water supplies, and its expected effect on international conflict and migration, the death rate attributable to AGW (the difference between the two lines) may approach 10^7^ per year by 2100. If so, the total AGW death toll in the 21st century will be 5 × 10^8^ (the area between the two lines). Assuming another 5 × 10^8^ climate deaths in the 22nd and later centuries, the total death toll due to historic and current emissions will be roughly 10^9^.

## The 1,000-Tonne Rule

If burning 10^12^ tonnes of fossil carbon causes the premature deaths of 10^9^ future people, one future person is killed every time a 1,000 tonnes of carbon are burned. This OME is consistent with Nolt’s (2011a) finding that the average American produces 1,840 tonnes CO_2_ equivalent (from burning 500 tonnes of carbon) during her or his lifetime, which then causes the suffering or death of one or two future people. If it takes 1,000 tonnes of carbon (or 3,700 tonnes of CO_2_) to kill a future person, and Nolt’s calculation is correct, the average American is killing half of a future person.

The 1,000-tonne rule can be understood by the following thought experiment. Imagine what would happen if humanity suddenly and completely stopped producing GHGs. Today’s people would suddenly stop causing atmospheric GHG concentration to increase, which is killing future people. Relative to that scenario, the 1,000-tonne rule says that every additional 1,000 tonnes of carbon burned causes one future death. The rule predicts that the death will happen in the next 1–2 centuries, probably in a developing country. Beyond that, it says nothing about the time and place of death.

**Figure 3:**
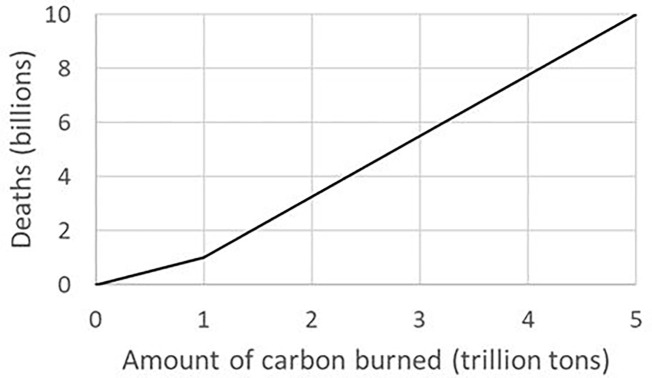
Predicted future global mortality as a function of carbon burned. Rough sketch of the assumed relationship. The 1,000-tonne rule applies to the left portion of the graph, before the first 10^12^ tonnes of carbon have been burned.

The horizontal axis of Figure 3 shows the total mass of carbon burned since the start of industrialization. The vertical axis shows the predicted number of future deaths due to AGW. This sketch is based on two assumptions: (1) burning 10^12^ tonnes of carbon will probably cause 10^9^ future premature deaths, and (2) burning 5 × 10^12^ tonnes of carbon will eventually kill most or all humans (10^10^ deaths). All values are OMEs. In the lower left corner, the assumed linear relationship between the number of future deaths caused and the mass of carbon burned corresponds to the 1,000-tonne rule. The concave-upward shape of the graph as a whole suggests that the amount of carbon to kill a future person is slowly decreasing.

Consider a medium-level “business as usual” scenario (e.g., IPCC Assessment Report 5, Working Group II, Representative Concentration Pathways) with a GMST increase of 3–4°C by 2100. If business as usual were continued into the future until all reasonably accessible carbon reserves (5 × 10^12^ tonnes according to Tokarska et al., 2016) were exhausted, GMST would increase by some 10°C, killing most or all humans (Sherwood and Huber, 2010; the right side of Figure 3). In that extreme case, roughly 500 tons of carbon will have been sufficient to kill one person.

## Avoiding Bias

Is it reasonable to predict that 10% of a future world population of 10^10^ will die prematurely following 2°C AGW? The prediction may be conservative for the following reasons:

1. The fatal future consequences of current human emissions have been assumed to last for 1–2 centuries; they may last for a millennium.
2. The 1,000-tonne rule has been derived on the assumption that AGW will double the global death rate in connection with poverty. The current death rate has been estimated at 10^7^ per year; but Pogge (2001) placed it at 1.8 × 10^7^.
3. The prediction relies on IPCC predictions, which tend to be conservative (Rahmstorf et al., 2007, 2012; Rahmstorf, 2010; Scherer, 2012; Brysse et al., 2013; Saba et al., 2016). Current ocean warming is at the high end of previous IPCC estimates (Resplandy et al., 2018), contributing to increases in rainfall intensity, sea level, and reef destruction, and decreases in ocean oxygen levels, mass of ice sheets, glaciers and ice caps (Cheng et al., 2019). Not all GMST models cited by IPCC have factored in the expected fall in carbon absorption rates by plants in the second half of the 21st century (Green et al., 2019). Conservative estimates may be a response to political and financial pressure from fossil fuel industries and the global climate denial community; climate scientists who publish the truth may even risk losing their jobs (Shogren, 2019).

If the present claims seem exaggerated relative to public current discourse on AGW, the reason may be denial in public discourse (Sandman, 2009)—a complex psychological, sociological, political, economic, and ethical phenomenon. Misleading claims by high-profile climate deniers (“contrarians,” “skeptics”) have appeared commonly in the media during the past two decades. Many non-experts still think AGW is one side of an unresolved discussion (Oreskes, 2004). Middle-class Westerners creatively justify their failure to reduce personal emissions, citing financial cost, the responsibility of others including governments, or the uncertain efficacy personal action (Stoll-Kleemann et al., 2001). Further “dragons of inaction” include limited knowledge, skepticism, non-ecological ideological worldviews, and risk underestimation (Gifford, 2011). Perceptions of climate risk depend strongly on psychosocial identity—whether political conservative or progressive, farmer, scientist, environmentalist, or businessperson (Fielding and Hornsey, 2016). Identity mediates the link between social representations of climate change and mitigating/adapting behaviors (Jaspal et al., 2014).

Any estimate of massive unprecedented risk is subject to psychological distortions (Yudkowsky, 2008), depending on “gender, political party, knowledge of the causes, impacts and responses to climate change, social norms, value orientations, affect and personal experience with extreme weather” (Van der Linden, 2015, p. 112). Humans tend to overestimate small risks and underestimate large risks (Hakes and Viscusi, 2004). For example, people living in earthquake zones tend to purchase inadequate insurance (Lehman and Taylor, 1987); “people refuse to buy flood insurance even when it is heavily subsidized and priced far below an actuarially fair value” (Yudkowsky, 2008, p. 92). The dangers to society and aviation posed by volcanic ash dispersal are typically underestimated (Bourne et al., 2016). Smokers underestimate the personal risk of smoking (Viscusi, 1990).

There is a similar tendency to underestimate risks based on description by comparison to those based on visceral experience (Weber, 2006). Unprecedented events are an example, and given that AGW will last for centuries, historical examples are relevant. In 1912, the Titanic provided 20 lifeboats for 1,178 people. In 1914, the Great Powers estimated that a modern war would be over in a few weeks. In 1939, following further improvements in military technology, the Nazis planned to win a two-front war. In 2003, Bush and Blair predicted their high-tech Iraq invasion would be fast and efficient with few casualties. Most economists failed to predict the 2008 financial crisis.

According to the *availability heuristic* (Tversky and Kahneman, 1973), people are more likely to judge an event as likely or frequent if instances are easy to imagine or recall. This gives people first-hand experiences of the event’s consequences (Spence et al., 2011. In the case of AGW, no such instances are available, because the situation is unprecedented and hindsight is not possible. “Risks of human extinction may tend to be underestimated since, obviously, humanity has never yet encountered an extinction event” (Yudkowsky, 2008, p. 93).

Many believe that AGW will be mitigated by technological advances (Hulme, 2014). Technology has solved many problems before, radically improving standards of living—in both rich and poor countries. But reliance on technology to solve future problems is an example of the logical fallacy of “hasty generalization.” Many technological solutions to AGW are possible, but all are subject to physical, social, and economic limitations (Huesemann, 2003). All currently known climate-engineering solutions have limited effectiveness and/or dangerous byproducts (Keller et al., 2014). Technology can hardly solve behavioral problems grounded in evolution such as xenophobia and selfishness that alone could lead to human self-destruction (cf. Persson and Savulescu, 2013).

Finally, a lack of positive active engagement with climate issues may cause individuals to underestimate the magnitude of the problem. Passivity may be promoted by public information that induces fear rather than “non-threatening imagery and icons that link to individuals’ everyday emotions and concerns” (O'Neill and Nicholson-Cole, 2009, p. 355).

## Semi-Quantitative Prediction

The predicted death rates are *semi-quantitative*, lying between qualitative and quantitative and relying on OMEs. An underlying assumption is that a very rough estimate is better than none at all. It is more informative to speak of “very roughly a million people,” and to base ethical, political, and economic arguments on such estimates, than merely to consider “a very large number of people.”

A semi-quantitative approach to ethics can clarify the magnitude and importance of AGW by comparison to other dangers. For example, since 2001 the threat of terrorism has influenced politics and policy more than the threat of climate change (Sunstein, 2007). In Western or richer countries, terrorists sometimes kill ten or a hundred people, attracting extensive media coverage. While every such attack is a tragedy, it is small compared to other cases of violence. The ongoing crisis in Iraq receives relatively little media attention despite the roughly 10,000 violent documented deaths every year since the 2003 US-UK invasion (Iraq Body Count, internet). Yet even that national tragedy is small compared to the human cost of poverty and AGW. The global number of under-five deaths per year decreased from over 1.2 × 10^7^ in 1990 to 7.6 × 10^6^ in 2010 (Pérez-Moreno et al., 2016). Between 5 and 14 years of age, 10^6^ children are dying yearly from mainly preventable causes (Fadel et al., 2019). The present analysis suggests that global GHG emissions are killing future people (mainly children) at a comparable rate.

## Conclusion

Convergent evidence from diverse approaches suggests that 2°C AGW, caused by burning 10^12^ tonnes of carbon (equivalent), will kill roughly 10^9^ people over a period of one to two centuries. Therefore, one future person is killed every time roughly 10^3^ tonnes of carbon are burned. This “1,000-tonne rule” implies that every fossil fuel industry whose productivity can be expressed in millions of tonnes of burned carbon is causing the premature deaths of thousands of future people. Consider the following examples:

1. A typical passenger jet carries 300,000 l of fuel and consumes 200,000 on a long flight, creating 500 tonnes of CO_2_ corresponding to 135 tonnes of carbon. That is about 1/8 of 1,000 tonnes, or 1/8 of a human life, according to the 1,000-tonne rule. Aircraft also emit other GHGs, and the high altitude at which the GHGs are emitted must also be considered. If the overall warming effect is at least twice the effect of the CO_2_ alone (Penner et al., 1999), a future person dies for every four long flights, on average. Therefore, flying should be made more expensive (e.g. by carbon taxes) and reserved for emergencies and life-saving projects.
2. Every year, Australia exports 4 × 10^8^ tonnes of coal. If that coal is 80% carbon, the country is exporting about 3 × 10^8^ tonnes of carbon per year. When that coal is burned, 3 × 10^5^ future deaths are caused every year. Clearly, this and comparable industries must be rapidly wound down to protect the rights of future generations. Employees can be retrained for the growing sustainable energy industry (Louie and Pearce, 2016).
3. The 1,000-tonne rule allows the 2015 Paris agreement to be newly interpreted as an agreement to limit the number of deaths caused by AGW to 10^9^. A statement of that kind has enormous political and ethical implications.

Quantitative mortality estimates allow policy decisions to draw more systematically on comparisons between AGW with other anthropogenic causes of premature death. Cigarettes currently kill 7 × 10^6^ people per year (Rentería et al., 2016). Comprehensive cigarette advertising bans can significantly reduce the incidence of smoking (Saffer and Chaloupka, 2000). GHG emissions are killing future people at a comparable or higher rate, and fossil fuel consumption has been likened to addiction (Suranovic, 2013). Therefore, advertising for fossil cars and distant holidays (implying flying) should be comprehensively banned.

The 1,000-tonne rule can help estimate energy risks and guide policy decisions in other ways. In 2014, world energy consumption comprised fossil fuels (86%), hydro/bioenergy/geothermal (8%), nuclear (4%), and wind/solar (2%) (Moriarty and Honnery, 2016). In 2017, the proportions were oil 34%, coal 28%, natural gas 23%, and renewables 4% (BP, 2018). What proportions would be sustainable in the 22nd century? The question may be reduced to one of mathematical optimization (cf. Nordhaus, 1994). A human-rights approach would minimize the number of lives lost per energy unit.

Also relevant is the question of whether nuclear energy is a reasonable alternative to fossil fuels in the anthropocene (Hansen et al., 2013b). The answer depends on predicted long-term mortality per unit energy for each source. For nuclear power, one might estimate the probability of different accident scenarios and the number of deaths caused by each, including scenarios in which nuclear wastes impact future generations. The nuclear industry estimated the cancer death toll from the Chernobyl meltdown in 1986 to be a few thousands, but it could have exceeded 10^5^ (Yablokov et al., 2010). Considering other nuclear accidents, nuclear energy may currently be causing roughly 10^5^ deaths globally per year (OME) by comparison to 10^7^ for fossil fuels. Fossil fuels, which are generating some 20 times more power than nuclear fission, may be causing 100 times (OME) more deaths, making them five times (OME) less efficient in this human-rights-oriented sense—consistent with Hansen’s claim. But renewables can also cause future deaths: the land used by solar power plants might otherwise be used for agriculture, alleviating future famines, and hydroelectricity affects biodiversity when rivers are dammed.

The main limitation of the present approach is uncertainty. All numerical estimates are assumed uncertain by plus or minus a few tens of percent—unavoidable, when extrapolating toward unprecedented future situations. But the explicit acknowledgment of uncertainty is also a strength. IPPC reports were improved by quantifying probabilities associated with qualifiers such as “likely” (>66%) or “virtually certain” (>99%) (IPCC, 2010).

Another limitation is the explicitly anthropocentric focus. Only human lives have been considered; the intrinsic value of other species (ecocentric and biocentric ethics; Nolt, 2011b) has been ignored. Deaths and extinctions of other species have been considered only relative to their effect on human mortality. A more ecocentric approach might have significant implications for conservation practice and policy (Kopnina et al., 2018). One might “take a pragmatic approach by which primary human needs are met first and foremost whereas the needs of other living organisms and ecosystems are allowed to prevail over secondary human needs” (Bourdeau, 2004, p. 9).

The question of economic growth versus steady-state economies (Buch-Hansen, 2014) has not been considered. In a cost-benefit analysis, the economic benefits of growth in the absence of environmental degradation may have fallen below the effective long-term environmental costs (the cost of inaction; Bosetti et al., 2009). But the dependence of future death tolls on carbon burned in the present analysis may be largely independent of economic context.

The possibility of future discounting (Dasgupta, 2008) has not been systematically considered, nor has the precautionary principle (Kriebel et al., 2001). These two principles would change predictions in opposite directions and partially cancel each other.

## Data Availability Statement

The raw data supporting the conclusions of this manuscript will be made available by the authors, without undue reservation, to any qualified researcher.

## Author Contributions

The author confirms being the sole contributor of this work and has approved it for publication.

### Conflict of Interest

The author declares that the research was conducted in the absence of any commercial or financial relationships that could be construed as a potential conflict of interest.

## Footnotes

^1^trillionthtonne.org

^2^worldometers.info
